# Ultrafine-Grained Tungsten Heavy Alloy Prepared by High-Pressure Spark Plasma Sintering

**DOI:** 10.3390/ma15176168

**Published:** 2022-09-05

**Authors:** Shuaihao Zhang, Qiqi Zhu, Qiunan Li, Wei Ji, Weimin Wang, Zhengyi Fu

**Affiliations:** 1 State Key Laboratory of Advanced Technology for Materials Synthesis and Processing, Wuhan University of Technology, Wuhan 430070, China; 2 Hanjiang Hongyuan Xiangyang Silicon Carbide Special Ceramics Co., Ltd., Xiangyang 441000, China

**Keywords:** tungsten heavy alloy, ultra-fine grain, high pressure, spark plasma sintering, hardness

## Abstract

Tungsten heavy alloy (WHA) is an ideal material employed for kinetic energy penetrators due to its high density and excellent mechanical properties. However, it is difficult to obtain ultrafine-grained tungsten alloy with excellent properties by traditional powder metallurgy method because of severe grain growth at a high sintering temperature with a long soaking time. In this study, the sintering behavior of tungsten alloys was studied at 800 to 1300 °C, and highly dense 93W-5.6Ni-1.4Fe (wt.%) WHA was successfully fabricated at a low temperature of 950 °C with a high pressure of 150 MPa by spark plasma sintering. The as-sintered tungsten alloy possesses a high relative density (98.6%), ultrafine grain size (271 nm) and high dislocation density (2.6 × 10^16^ m^−2^), which results in excellent properties such as a high hardness (1079 HV1). The high sintering pressure is considered to support an additional driving force for the sintering and lead to a low-temperature densification, which effectively limits grain growth.

## 1. Introduction

Tungsten heavy alloys (WHAs) are typical diphase composite materials, in which W element acts as the matrix and a small amount of other elements such as Ni, Cu, Fe, and Mo act as additives. These alloys are promising candidates for applications in kinetic energy penetrators, counter weights, anvil block materials, and facing plasma materials owing to their advantages, including a high density, high hardness and strength, good ductility, excellent corrosion and radiation resistance [1,2,3,4,5].

The fabrication methods for WHAs mainly include traditional liquid-phase sintering (LPS) [6,7,8], microwave-assisted sintering (MAS) [9,10,11,12,13], oscillating pressure sintering (OPS) [14], selective laser melting (SLM) [15], hot isostatic pressing (HIP) [16], and spark plasma sintering (SPS) [17,18,19,20,21]. LPS requires a high temperature with a long dwelling time to achieve a high density. During the process, due to the rapid mass transfer in the liquid phase, the grain of the WHAs grows much larger than that of the raw powder. SPS is considered to be favorable for preparing fine alloys owing to the fast heating rate, extremely short sintering time, and low sintering temperature, which could effectively inhibit grain growth.

The mechanical properties of alloy are affected by many factors, such as the characteristics of raw powder (grain size, W content and Ni: Fe ratio), different additives, and the annealing process [3,22]. It was revealed that nano-crystalline powders are beneficial for sintering. In addition, the fine-grained tungsten alloys could possess better dynamic mechanical properties than conventionally sintered alloys with coarse grains [23]. Different W contents and Ni: Fe ratios also affect the mechanical properties. WHAs with a higher W content and larger Ni: Fe ratio are suitable for kinetic energy penetrators [24]. According to Li’s work [18] and much other available literature, 93W-5.6Ni-1.4Fe heavy alloys could possess high mechanical properties. However, it is still a challenge to fabricate high relative density and fine grain bulks, which are expected to show an enhanced performance. Appropriate additives, such as Cr, Co, Mn, Re, Al, Si, Sn, Zn, YSZ, and Y_2_O_3_, were suggested to refine the microstructure of tungsten alloys [8,25,26,27,28,29,30]. The alloys could also be enhanced by cold plastic deformation after annealing at lower temperatures [31]. One of the most effective and direct ways to improve the mechanical properties of WHAs is grain refining. The Vickers hardness follows the Hall-Petch relationship [32,33]:

(1) $$H=H_{0}+{\mathrm{kd}}^{-1/2}$$

where *H*_0_ is the primary hardness of the tungsten alloy, *d* is the average grain size, and *k* is the Hall-Petch coefficient.

In order to obtain ultrafine-grained tungsten alloy, the sintering temperature should be limited to remaining below the critical temperature (T_sg_) at which the grains start to grow rapidly. However, the relative density is usually very low at this temperature. The application of pressure may help solve this problem. For many high-melting-point materials such as ceramics, it has been revealed that a high pressure could significantly reduce the sintering temperature and thus obtain dense materials with fine grains. Xu et al. prepared high density nano-sized Al_2_O_3_ with a grain size of 290 nm at 1050 °C by spark plasma sintering method under 200 MPa with limited grain growth [34]. Liu reported that nearly fully dense nanocrystalline MgO could be rapidly fabricated at 1350 °C and 170 MPa by combustion reaction and quick pressing method (CR-QP) [35]. Ji and co-workers successfully prepared nanocrystalline 3YSZ ceramics at 900 °C under 1.5 GPa using a piston-cylinder apparatus [36]. Thus, it is expected that ultrafine-grained tungsten alloy could be fully densified under a high pressure at a low temperature.

In this study, high-pressure spark plasma sintering technology (HPSPS) with an axial pressure of up to 150 MPa was firstly applied to fabricate ultrafine-grained 93W-5.6Ni-1.4Fe tungsten alloy with a high density and hardness at a low temperature below 1000 °C. The phase composition, densification behavior, microstructure evolution and mechanical properties were well-studied.

## 2. Experimental Materials and Methods

**Figure 1:**
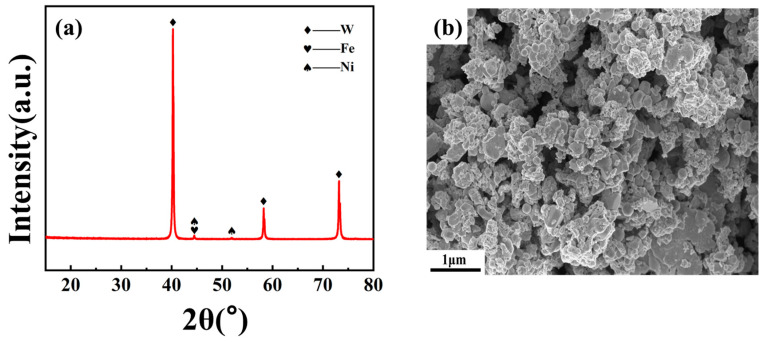
(**a**) XRD pattern and (**b**) SEM image of the mixed powders.

High-purity tungsten (W), nickel (Ni) and iron (Fe) powders were used as the raw materials (Beijing Zhongnuo Advanced Materials Technology Co., Ltd., Beijing, China), and their related physical properties are shown in Table 1. The elemental powders of W, Ni and Fe were mixed in a mass ratio of 93: 5.6: 1.4 and milled in a planetary ball miller (QX-QM-4L, Changsha Tianchuang Powder Technology Co., Ltd., Changsha, China) for 6 h at 300 rpm in vacuum. Tungsten carbide (WC) vials, balls and ethanol were utilized as the milling media in a ball-to-powder mass ratio of 10:1. The phase composition and morphology of the mixed powders are shown in Figure 1.

The tungsten alloys were fabricated by a spark plasma sintering apparatus (SPS, Elenix, Ed-pas Ⅲ, Zama-shi. Japan) using a carbon-fiber-reinforced carbon composite (C_f_/C) mold with an inner diameter of 12 mm, at temperatures of 800–1300 °C, with a uniaxial pressure of 150 MPa, soaking for 5 min. The C_f_/C mold was woven by carbon fiber in three dimensions, and it has a high strength at high temperatures. A low pressure of 50 MPa was also selected as the control sample. The temperature was automatically raised to 600 °C over a period of 3 min and was subsequently monitored and regulated by an infrared thermometer focusing on the die with a heating rate of 100 °C/min. During sintering, the die was wrapped with graphite wool for temperature homogeneity. The uniaxial pressure was applied before sintering and maintained during the rest of the heating and soaking time. Natural cooling began when the power was turned off at the end of the soaking, and the applied pressure was removed at the same time.

After sintering, the specimens were grinded and polished to remove surface impurities. In the processing of the grinding, after the metal surface of specimens is completely exposed, continue grinding by 2 mm. The relative density was measured by Archimedes’ method. The phase composition was detected by X-ray diffraction (XRD, Empyrean series 3, Malvern Panalytical, Eindhoven, The Netherlands) using Cu Kα radiation (λ = 1.540598 Å) from 30° to 90°. A scanning electron microscope (SEM, GeminiSEM 300, ZEISS, Oberkochen, Germany) was used to observe the microstructure of the samples. Electron back-scatter diffraction (EBSD, Oxford Instruments, nordlysnano, Oxford, UK) was operated to measure the grain size of the as-preserved samples by line-intercept method using a three-dimension calibration factor of 1.571. The microstructure and grain boundary characteristics of the as-sintered samples were investigated in detail via a high-resolution transmission electron microscopy (HRTEM, Tecnai G2 F30, FEI, Portland, OR, USA). An energy dispersive spectrometer (EDS, Genesis, EDAX, Pleasanton, CA, USA) was applied to detect the element distribution of the samples. The hardness was measured by a Vickers hardness tester (Wolpert 430SVD, Boston, MA, USA) with an applied load of 1 Kgf for 15 s. The indentation was measured to calculate the hardness by [37]:

(2) $$\mathrm{HV}=0.102\;\times \frac{F}{S}=0.102\;\times \;\frac{2\mathrm{Fsin}\frac{\alpha }{2}}{d^{2}}$$

where *F* is the applied pressure, *α* is the angle (136°) between the indenter head, and *d* is the average diagonal length. The reported values of hardness are the average of at least ten measurements in the same relative location for the different samples.

## 3. Results and Discussion

### 3.1. Phase, Microstructure, and Densification of the Tungsten Alloys

**Figure 2:**
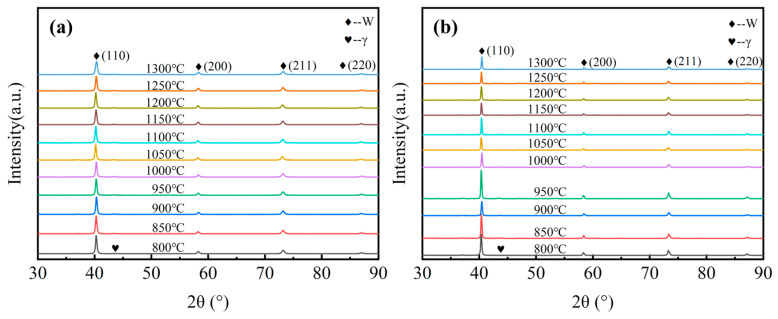
XRD patterns of 93W-5.6Ni-1.4Fe tungsten alloys after SPS at 800–1300 °C under (**a**) 50 MPa and (**b**) 150 MPa.

Figure 2 shows the XRD patterns of tungsten alloys sintered under different pressures and temperatures. According to the XRD images, the W phase and γ- (Ni, Fe, W) phase are mainly found in the tungsten alloys. The W phase is the main phase, and the minor γ phase acts as the binding phase. No further phase transformation of the alloy could be observed by XRD.

**Figure 3:**
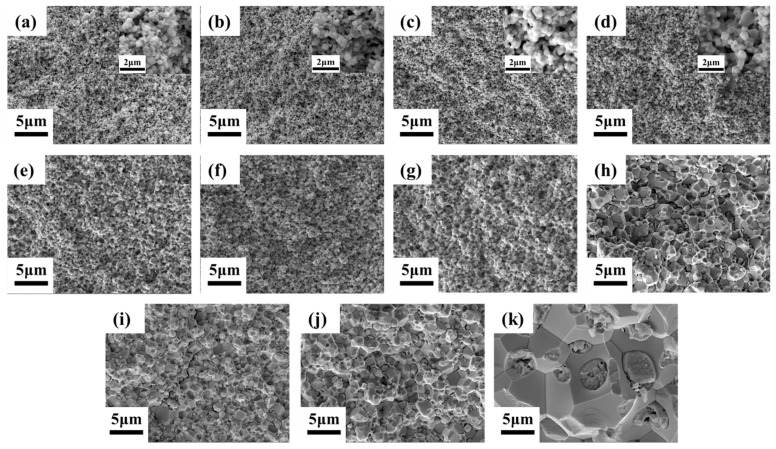
The fracture morphologies of 93W-5.6Ni-1.4Fe tungsten alloys after SPS under 50 MPa at different temperatures: (**a**) 800 °C, (**b**) 850 °C, (**c**) 900 °C, (**d**) 950 °C, (**e**) 1000 °C, (**f**) 1050 °C, (**g**) 1100 °C, (**h**) 1150 °C, (**i**) 1200 °C, (**j**) 1250 °C, and (**k**) 1300 °C. Inserts are the magnified images.

The SEM images of the fracture morphology of WHAs sintered at different temperatures under 50 MPa are shown in Figure 3. It could be obviously observed that when the sintering temperature was relatively low, the grain size was almost unchanged, after which the grain size increased slightly at ~1050 °C. When the temperature was elevated to above 1100 °C, the grains grew rapidly. The temperature also has a great influence on the density of WHAs. The sintering necks between particles formed at ~900 °C, which means the beginning of visible mass transfer and sintering. The alloys achieved a high relative density at 1050 °C, and there was almost no further improvement at higher temperatures. When the sintering temperature was higher than 1100 °C, tiny cracks appeared in the alloy, which caused a slight decrease in the relative density. The fracture mode of tungsten alloy is mainly intergranular fracture.

**Figure 4:**
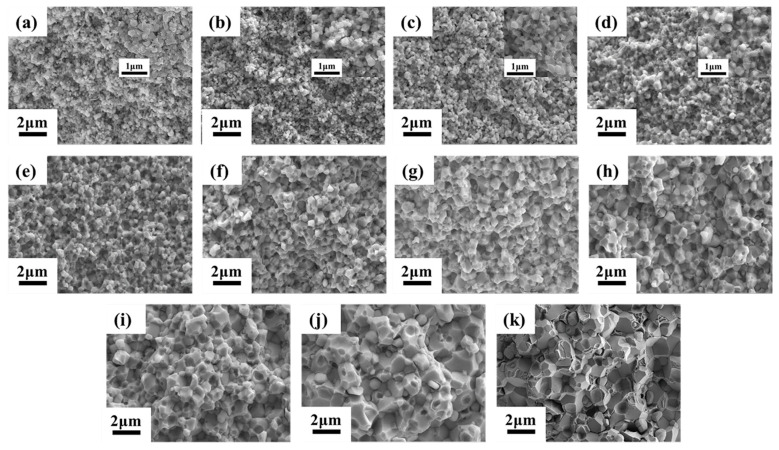
The fracture morphologies of 93W-5.6Ni-1.4Fe tungsten alloys after SPS under 150 MPa at different temperatures: (**a**) 800 °C, (**b**) 850 °C, (**c**) 900 °C, (**d**) 950 °C, (**e**) 1000 °C, (**f**) 1050 °C, (**g**) 1100 °C, (**h**) 1150 °C, (**i**) 1200 °C, (**j**) 1250 °C, and (**k**) 1300 °C. Inserts are the magnified images.

The fracture morphologies of alloys sintered at different temperatures under 150 MPa are shown in Figure 4. It was clearly indicated that the increase in the sintering temperature also led to grain growth, but the overall grain size was much smaller than for the samples sintered under 50 MPa. Sintering necks formed in the compact at 900 °C. At the higher temperature, mass transfer became faster. A high density could be reached at 950 °C. The main fracture mode of tungsten alloy is intergranular fracture. With the increase of the temperature, more bond phase tears appear, which is associated with the liquid phase produced at a high temperature.

**Figure 5:**
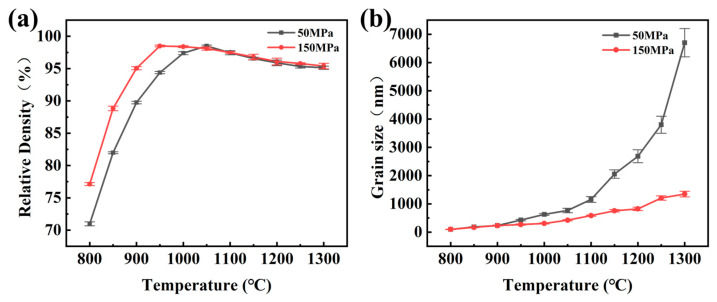
Change in (**a**) relative density and (**b**) grain size of 93W-5.6Ni-1.4Fe tungsten alloys sintered at 800–1300 °C under different pressures.

Figure 5a presents the densification curve of tungsten alloys under different pressures and temperatures. It can be seen that the relative densities of tungsten alloy sintered under 50 MPa and 150 MPa both first increase and then decrease with the rise in the sintering temperature. Specifically, the maximum relative density of tungsten alloy sintered under 150 MPa reaches 98.6% at 950 °C. However, the sintering temperature required in order for the sample sintered under 50 MPa to reach a relative density of 98.6% rose to 1050 °C. This is because the higher pressure provides a stronger driving force for densification, so that the tungsten alloy could attain full density at a much lower sintering temperature [34]. In addition, the high pressure could directly affect the rearrangement of powder particles and destroy the agglomeration in the powder compact, which is also beneficial to sintering [38]. With the rising of the sintering temperature, it is possible that a high local temperature may cause an overburning phenomenon for the γ phase. At the same time, an unbalanced shrinkage will occur during the rapid cooling and solidification process and produce a small shrinkage cavity, thereby leading to the decrease of the relative density at 1300 °C under both pressures [24]. This is a little different to the consolidation process of tungsten alloy prepared by traditional liquid-phase sintering.

Figure 5b shows the grain growth of tungsten alloy with an increasing temperature under both pressures. At each temperature point, the grain size of tungsten alloy under 50 MPa is obviously larger than that of the samples under 150 MPa. From 800 °C to 1000 °C, the grain size of the samples increases slowly from 100 nm to 628 nm (with 50 MPa) and 309 nm (with 150 MPa), respectively. When the temperature exceeds 1050 °C, the grains grow rapidly, reaching 6.67 μm (with 50 MPa) and 1.35 μm (with 150 MPa) at 1300 °C, respectively [39]. The Ostwald maturation theory is generally used to explain the grain growth of tungsten alloys during sintering. When the small grains dissolve in the W matrix, the W matrix reaches the supersaturation state. The solute in the matrix precipitates around the large grains, which leads to the ablation of the small grains and the growth of the large grains. As a result, the average grain size increases [6].

### 3.2. EBSD and TEM Analysis of the Tungsten Alloys

**Figure 6:**
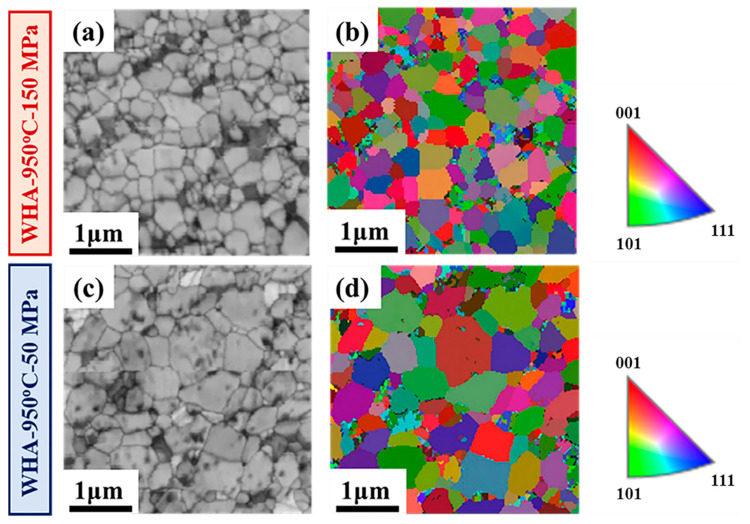
The EBSD results of band contrast images and Inverse Pole Figure (IPF) images for tungsten alloys sintered at 950 °C under (**a**,**b**) 150 MPa and (**c**,**d**) 50 MPa, respectively.

Figure 6 displays the EBSD images of tungsten alloys sintered at 950 °C under different pressures. It can be seen that the grain sizes of both tungsten alloys are uniform and that the shape is an irregular polygon. The preferred orientation is not observed. It is obvious that the grain size of the sample sintered under 150 MPa is much smaller than that under 50 MPa. The results indicated that grain growth could be restrained by high pressure. Figure 6a,c present almost no pores and cracks, which indicated the high density of tungsten alloys.

**Figure 7:**
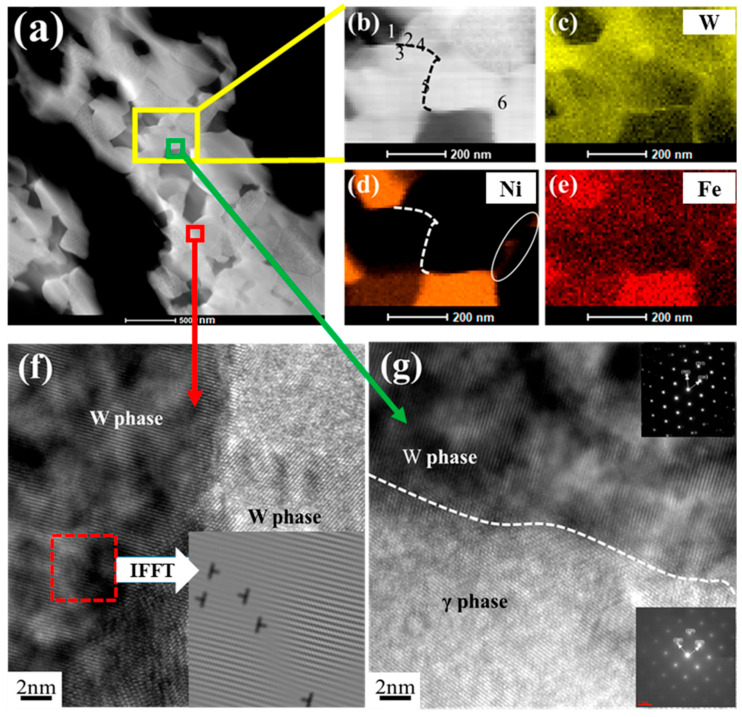
TEM images of 93W-5.6Ni-1.4Fe alloy sintered at 950 °C under 150 MPa. (**a**,**b**) Dark field image of WHA; The element distribution of (**c**) W, (**d**) Ni and (**e**) Fe; The HRTEM images of grain boundaries within (**f**) W grains and between (**g**) W and γ phases.

Figure 7 gives the detailed microstructure of 93W-5.6Ni-1.4Fe alloy sintered at 950 °C under 150 MPa. The dark field image in Figure 7a shows that the sample is mainly composed of bright and dark phases. The mapping images in Figure 7b–e manifest the distribution of W, Ni, and Fe. The bright phase is a W-enriched phase and the dark phase is a (Ni, Fe)-riched phase. The composition analysis results in Table 2 demonstrate that a small amount of Ni and Fe enters the tungsten lattice to form a solid solution, resulting in lattice distortion. The bright region contains 97 wt.%-99 wt.% of W elements. In addition, about 27 wt.% of W elements dissolved in the dark phase.

There are two kinds of connection types for W grains in the as-sintered alloy. One is the direct connection among W grains by diffusion (Figure 7f), which is solid phase sintering. The other one is bonded by the γ phase, which exists in the gap between irregular tungsten grains, as shown in Figure 7d,g. Figure 7f shows the dislocations of the W phase. The dislocation density could be calculated by [40]:

(3) $$\rho =\frac{L}{V}=\frac{\mathrm{nl}}{\mathrm{lA}}=\frac{n}{A}$$

where Ais the selected area, and n is the number of dislocations existing in A. According to the equation, the density of dislocation in Figure 7f is 2.6 × 10^16^ m^−2^. The high-pressure sintered tungsten alloy has reached the work-hardening state. The movement of dislocations will be hindered by the high density of dislocations, which could enhance the resistance to deformation and promote the mechanical properties.

### 3.3. Mechanical Properties of the Tungsten Alloys

**Figure 8:**
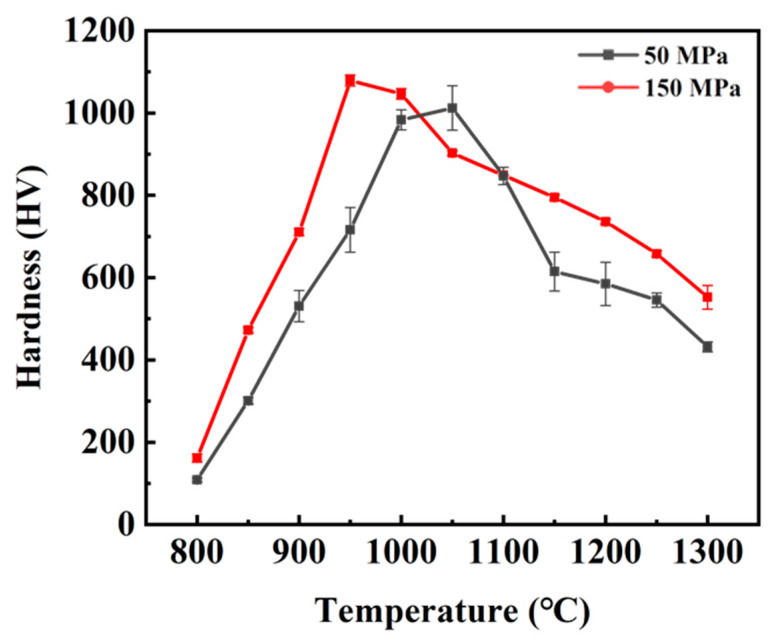
The relationship between sintering temperature and hardness of 93W-5.6Ni-1.4Fe tungsten alloys sintered under different pressures.

Figure 8 describes the Vickers hardness of tungsten alloys sintered at different temperatures under 50 MPa and 150 MPa, respectively. With the increase of the sintering temperature, the Vickers hardness increases initially and then decreases under both pressures, which is consistent with the change in relative density. In addition, the decreasing trend after the peak value is much more dramatic. The hardness of the tungsten alloys sintered by SPS under 50 MPa increased from 99.46 HV at 800 °C to 927.84 HV at 1150 °C, and then decreased to 395.84 HV at 1300 °C. As for the specimen sintered under 150 MPa, the hardness increased from 161.77 HV at 800 °C to 1079 HV at 950 °C, and decreased to 552.37 HV at 1300 °C. The reported results on the relative density, grain size and hardness by different consolidation technologies are listed in Table 3. The ultrafine-grained alloy fabricated by HPSPS in the present work shows one of the highest hardness values reported.

The reasons for this phenomenon are considered to be as follows. The hardness of tungsten alloys increases with the densification, while it decreases with grain growth. In addition, the deformation of metal is mainly caused by dislocation slipping and climbing [44]. The dislocation movement can be hindered by the grain boundaries and a high dislocation density. According to the Hall-Petch relationship, the smaller the grain size, the more grain boundaries there are, and the better the performance is.

When the temperature is relatively low, the density gradually increases, while the grain size does not grow much. Thus, the density plays a dominant role in the mechanical properties. In the present work, the hardness reaches the maximum due to the high density, small grain size and high dislocation density at a critical temperature. After that, the relative density decreases slightly, while the grain grows rapidly, resulting in a dramatical degradation of the mechanical properties.

## 4. Conclusions

1. Fully dense 93W-5.6Ni-1.4Fe tungsten alloy with an ultrafine grain size of 271 nm, high relative density of 98.6% and high dislocation density of 2.6 × 10^16^ m^−2^ was successfully fabricated by spark plasma sintering under a high pressure of 150 MPa and a low temperature of 950 °C.
2. The as-sintered alloy includes a W-enriched phase and a γ-bonded phase. With the increase of the sintering temperature, no further phase transformation occurred, while the relative density and mechanical properties first increased and then decreased.
3. High pressure could provide a larger driving force for densification. Therefore, the tungsten alloys could be fully densified at a low temperature with limited grain growth. At the same time, high pressure could also lead to a high dislocation density, collectively leading to a high hardness.

## Figures and Tables

**Table 1:** Characteristics of powders used to prepare WHAs.

Element	Tungsten	Nickel	Iron
Particle size (μm)	0.1	0.1	0.1
Purity (wt%)	≥99.95%	≥99.95%	≥99.95%
Particle shape	Polyhedral	Spherical	Spherical

**Table 2:** EDS composition analysis results of spots 1 to 6 in Figure 7b.

	W (wt%)	Ni (wt%)	Fe (wt%)
Spot 1	27.07	71.07	1.84
Spot 2	97.32	2.67	0.00
Spot 3	97.35	1.10	1.54
Spot 4	96.77	3.01	0.20
Spot 5	97.46	1.83	0.69
Spot 6	98.63	1.36	0.00

**Table 3:** Physical and mechanical properties of 93W-5.6Ni-1.4Fe alloys prepared in this study compared with those from the literature.

Powder Size (μm)	Processing Methods	Relative Density (%)	Temperature (°C)	Hardness (HV)	Grain Size (μm)	Reference
1~3	Microwave ^a,^*	98.6	1500	410	-	[10]
18	SLM ^b,^*	96.1	-	-	-	[15]
3	OPS-HP ^c,^*	95	1250	450	3.8	[14]
20	LPS ^d,^*	97.1	1500	294	22.7	[41]
2.5	SPS ^e,^*	99.4	1410	-	-	[42]
2.3~2.7	SPS	94.7	1100	430	3~5	[20]
1~3	SPS	90	1000	746	1.5	[43]
0.1	SPS	98.1	1050	850	0.87	[19]
**0.1**	**SPS-150 MPa**	**98.6**	**950**	**1079 ± 14**	**0.27**	**Present work**

^a,^* Microwave-assisted heating; ^b,^* Selective laser melting; ^c,^* Oscillating pressure sintering-hot pressure; ^d,^* Liquid phase sintering; ^e,^* Spark plasma sintering.

## Data Availability

Not applicable.
